# P-1056. Advancing sterilization practices: the impact of a newly developed hydrogen peroxide vaporization robot system compared to traditional methods

**DOI:** 10.1093/ofid/ofaf695.1251

**Published:** 2026-01-11

**Authors:** Jung-wan Park, Su Ha Han, Jung-Eun Yu

**Affiliations:** Division of Infectious Diseases, Department of Internal Medicine, Soonchunhyang University Cheonan Hospital, Cheonan, Korea, Cheonan, Ch'ungch'ong-namdo, Republic of Korea; Soonchunhyang University College of Medicine, Cheonan, Ch'ungch'ong-bukto, Republic of Korea; Soonchunhyang university hospital, Cheonan, Cheonan, Ch'ungch'ong-namdo, Republic of Korea

## Abstract

**Background:**

Infection prevention and control (IPC) in healthcare settings is crucial for minimizing healthcare-associated infections (HAIs). Hydrogen peroxide vaporization (HPV) is widely used for surface disinfection; however, conventional methods require manual setup and suffer from inconsistent vapor distribution. This study evaluates a newly developed HPV Smart Robot system designed to automate and optimize disinfection, ensuring uniform hydrogen peroxide distribution while reducing human involvement and exposure.

**Figure:**
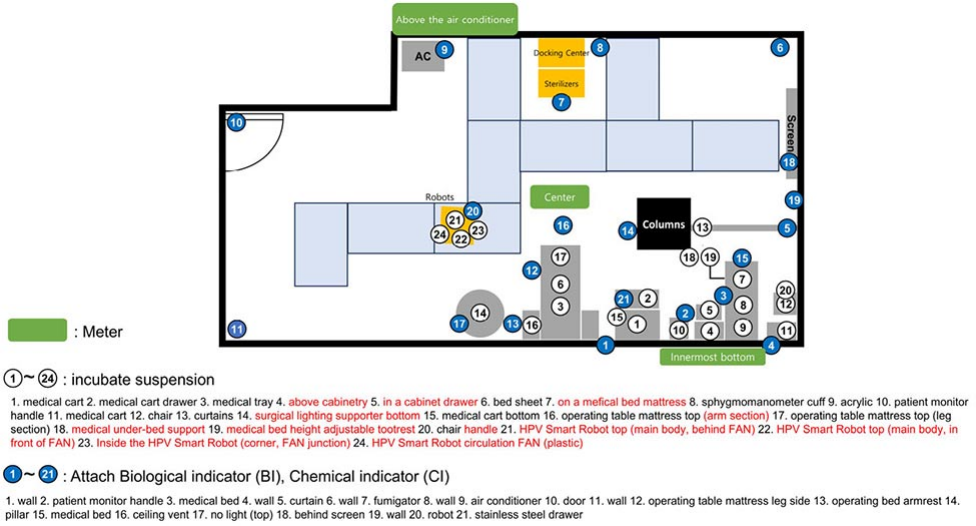
Infectious disease medical device clinical trial center structure, Geobacillus stearothermophilus application locations, Biological indicator, chemical indicator attachment location, and hydrogen peroxide concentration meter location

**Figure:**
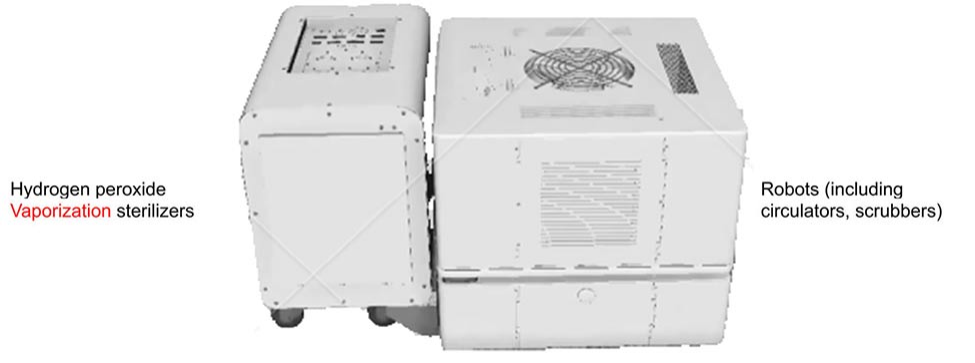
The HPV Smart Robot (Hydrogen Peroxide Vaporization Smart Robot system)

**Methods:**

A controlled experimental study was conducted in a clinical simulation center to compare the sterilization efficacy of the HPV Smart Robot with conventional HPV disinfection. Standard spores of *Geobacillus stearothermophilus* were inoculated on various surfaces, and post-sterilization bacterial cultures were analyzed. Hydrogen peroxide concentration, distribution uniformity, and aeration efficiency were assessed using biological and chemical indicators.

**Figure:**
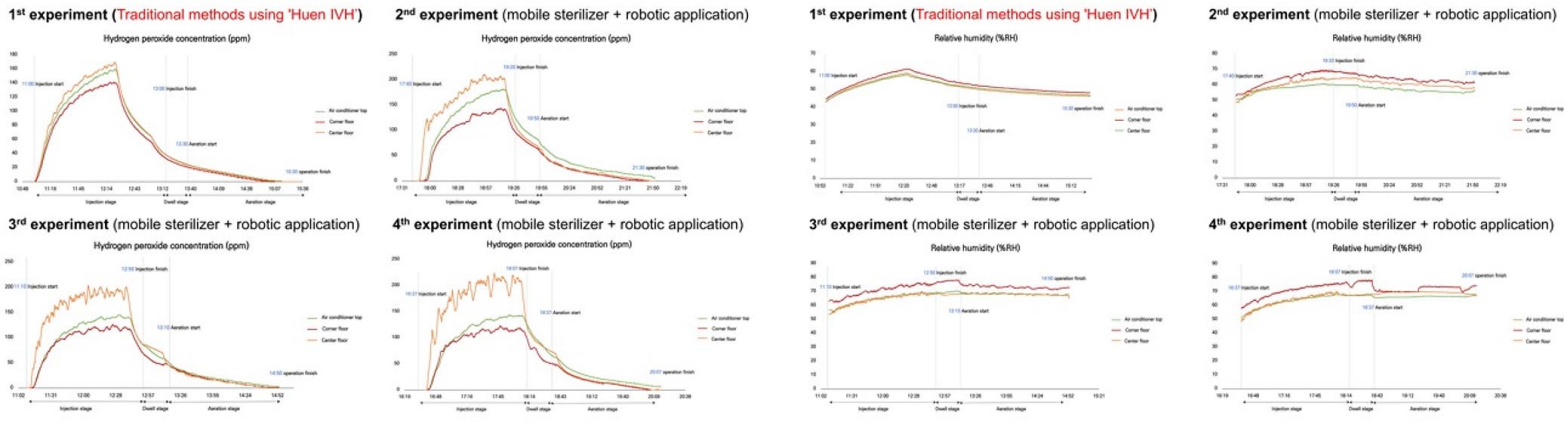
Hydrogen peroxide distribution concentration change and humidity change (a) Change in hydrogen peroxide distribution concentration. (b) Humidity changes in the clinical trial center

**Figure:**
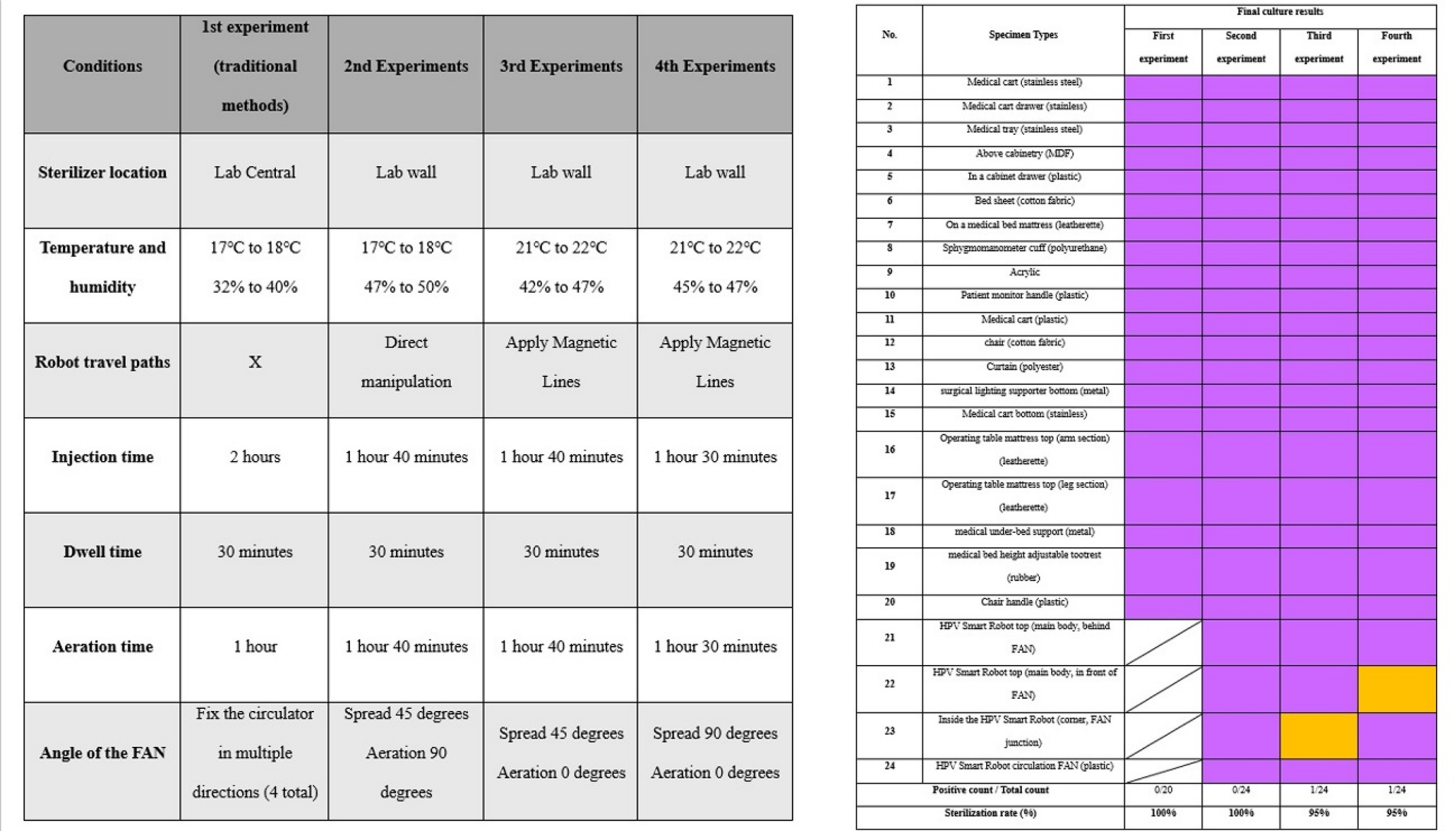
Experimental Conditions and Sterilization effect Positive (Bacteria detected) : purple color Negative (No bacteria detected) : orange color

**Results:**

Both conventional and robotic HPV disinfection achieved complete microbial inactivation, with all biological indicators confirming sterilization. The HPV Smart Robot demonstrated superior vapor distribution but showed a higher variability in peak hydrogen peroxide concentrations. Additionally, the robotic system reduced aeration time by 20%, minimizing operational downtime.

**Conclusion:**

The HPV Smart Robot effectively automates the sterilization process, reducing manual labor and exposure risks while maintaining comparable disinfection efficacy to traditional methods. Despite minor inconsistencies in vapor concentration, the system offers promising advantages in IPC strategies. Future research should explore real-world clinical implementation and cost-effectiveness to enhance infection control measures in healthcare environments.

**Disclosures:**

All Authors: No reported disclosures

